# Pharmacokinetic analysis of topical tobramycin in equine tears by automated immunoassay

**DOI:** 10.1186/1746-6148-8-141

**Published:** 2012-08-21

**Authors:** Sarah L Czerwinski, Andrew W Lyon, Brian Skorobohach, Renaud Léguillette

**Affiliations:** 1 Department of Veterinary Clinical and Diagnostic Sciences, Faculty of Veterinary Medicine, University of Calgary, 3330 Hospital Drive NW, Calgary, AB, T2N 4N1, Canada; 2 Department of Pathology and Laboratory Medicine, Faculty of Medicine, University of Calgary, Calgary, AB, Canada; 3 Calgary Laboratory Services, Calgary, AB, Canada; 4 Calgary Animal Referral and Emergency Centre, Calgary, AB, Canada

**Keywords:** Antibiotic, Equine, Pharmacokinetics, Tear, Tobramycin, Topical, Ophthalmology

## Abstract

**Background:**

Ophthalmic antibiotic therapy in large animals is often used empirically because of the lack of pharmacokinetics studies. The purpose of the study was to determine the pharmacokinetics of topical tobramycin 0.3% ophthalmic solution in the tears of normal horses using an automated immunoassay analysis.

**Results:**

The mean tobramycin concentrations in the tears at 5, 10, 15, 30 minutes and 1, 2, 4, 6 hours after administration were 759 (±414), 489 (±237), 346 (±227), 147 (±264), 27.6 (±28.4), 14.8 (±66.6), 6.7 (±18.6), and 23.4 (±73.4) mg/L. Mean tobramycin concentration was maintained above the MIC_90_ for commonly isolated bacteria for 68.5 min.

**Conclusion:**

A single dose of topical tobramycin resulted in therapeutic concentrations of tobramycin in the tears for 1 h after administration. Therapeutic levels of tobramycin remained in equine tears 6 times longer than was reported in rabbit tears.

## Background

Ulcerative keratitis commonly occurs in horses. Their prominent eye profile and active nature likely predispose them to ocular trauma [1]. Breaks in the corneal surface allow commensal conjunctival and corneal organisms to colonize the epithelium or the stroma. Primary corneal bacterial pathogens, such as *Pseudomonas,* can establish infection despite an intact corneal epithelium [1-3]. Bacteria most frequently cultured from the corneas of horses with ulcerative keratitis include *Streptococcus equi* subspecies *zooepidemicus*, *Pseudomonas aeruginosa*, and *Staphylococcus* spp. [2,4,5].

Although general recommendations are to use a broad-spectrum initial antimicrobial therapy for ulcerative keratitis, the antibiotic choice and frequency of administration are empirical and guided by the clinical examination and cytological evaluation of corneal swabs [1]. Therapy is modified based on bacterial and fungal culture and sensitivities, when they become available in the following days to weeks [1,6].

Appropriate antimicrobials to prevent infection include triple antibiotic ointment (neomycin, polymyxin B, bacitracin), tetracyclines, and aminoglycosides [1,2]. Fluoroquinolones are often used to treat established infections [1]. Treatment intervals for ulcerative keratitis range from q.6 h to q.8 h for prophylaxis of infection, and may be as frequent as q.2 h to q.4 h for corneas that are infected [1].

Tobramycin is commonly used to treat bacterial keratitis in horses. It is an aminoglycoside antibiotic with a mainly gram negative spectrum of activity. It is bactericidal, inhibiting protein synthesis through irreversible binding to the 30S and 50S ribosomal subunits [7]. To the authors’ knowledge, the pharmacokinetics of topical ophthalmic gentamicin and tobramycin has only been studied in humans and rabbits while the pharmacokinetics of ciprofloxacin has also been studied in horses and dogs [8-11]. In a rabbit study, tobramycin and ofloxacin concentrations were above the MIC_90_ for gram positive and gram negative organisms for 10 and 240 minutes, respectively, after drug administration, whereas gentamicin concentrations were above the MIC_90_ for 20 minutes for gram positive organisms and 120 minutes for gram negative organisms [10,12]. Another study done on humans found that after repeated administration, tobramycin and ofloxacin concentrations were above the mean MIC_90_ for 5 species of bacteria for 251 and 605 minutes respectively [11]. The pharmacokinetics of topical ophthalmic ciprofloxacin has been evaluated in the tears of normal horses and dogs. The ciprofloxacin concentrations remained above the MIC_90_ for 6 hours after administration in both species [8,9]. In all studies the antibiotic concentrations rapidly declined following administration [8-11].

Because tobramycin is frequently used empirically for the treatment of ocular disease in the horse, the objective of this study is to determine the pharmacokinetics of tobramycin in normal horses’ tears. We have measured the concentration of tobramycin in equine tears at varying time intervals following topical application.

## Materials and methods

The study was approved by the University of Calgary Veterinary Sciences Animal Care Committee. Ten healthy, mixed breed horses with normal ophthalmic examinations (including Schirmer tear test, slit-lamp biomicroscopy, indirect ophthalmoscopy, and fluorescein test) were used. There were 5 geldings and 5 mares, ranging in age from 1 to about 20 years old. Horses were housed in stalls for 24 hours prior to testing to prevent exposure to sun, wind, and dust.

Horses were sedated with xylazine intravenously (0.3 to 0.45 mg/ kg) to facilitate administration of the tobramycin. One fractious horse received butorphanol (0.02 mg/kg) in addition to xylazine prior to the administration of tobramycin. A 1 mL syringe and metal cannula was used to deliver 100 μL of 0.3% tobramycin (Tobrex®, Alcon Laboratories Inc., Fort Worth, TX, USA) into the ventral cul-de-sac of each eye on day 0. Tear samples were obtained by placing a pre-weighed Schirmer tear test strip in the ventral cul-de-sac of the eye for 30 seconds at 5, 10, 15, 30 minutes or 1, 2, 4, 6 hours after tobramycin administration. After tear collection the test strips were immediately weighed to determine the fluid weight collected and then stored in sealed 3 ml plastic tubes at −70°C until analysis. Tear volume in microliters was equated to tear weight in milligrams. To increase the number of samples per time point, both the left and right eyes of the 10 animals were treated on day 0, then once again after a seven day washout period. The sample times for the total 40 eyes were randomized, yielding 5 samples for each of the 8 time periods.

Tobramycin concentrations were determined by immunoassay. Schirmer test strips were extracted with 200 μl of drug-free human serum (Bio-Rad Laboratories Ltd, Mississauga, ON, Canada) for 10 minutes. The paper was mechanically pulped and then centrifuged at 10,000 g for 10 minutes. Supernatant fluid was analyzed on a Cobas C601 automated chemistry analyzer (Roche Diagnostics, Laval, Canada) using the manufacturer’s calibrators and serum tobramycin method at Foothills Medical Center, Calgary, AB, Canada. Evaluation of the extraction efficiency, precision and limit of detection for tear tobramycin from Schirmer test strips is described. Tobramycin concentration in the tears is expressed as mean ± SD. Pharmacokinetic analysis was performed using statistical software Stata 10 (College Station, TX, USA).

## Results

### Extraction and Immunoassay

**Figure 1:**
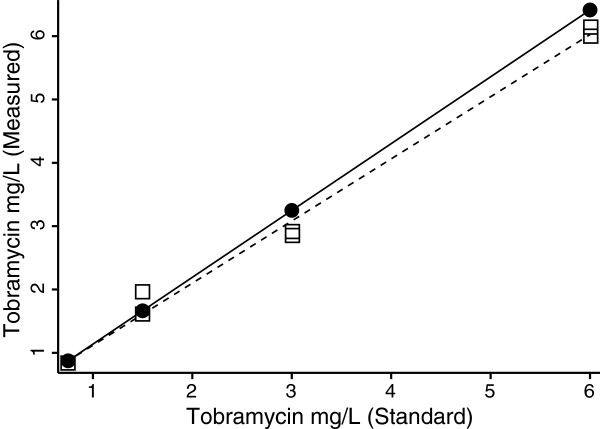
**Validation of the tobramycin immunoassay on tear samples:** Recovery of tear tobramycin from Schirmer test strips was assessed at four concentrations within the linear range of the immunoassay (0.5 – 10 mg/L) and compared to direct assay of dilute tobramycin solutions. The average recovery was 96.5%. Assays were performed in duplicated and least squares linear regression was used to derive the lines of best fit.

A tobramycin immunoassay developed for use on human serum was adapted for use with tear fluid collected on Schirmer test strips. In human serum, the assay performance specification was confirmed to be linear from 0.5 to 10 mg/L with within–day coefficients of variation of 3.6% at 1.7 mg/L and 3.5% at 7.8 mg/L (n = 10). The therapeutic Tobrex® (Alcon Laboratories Inc., Fort Worth, TX, USA) 0.3% solution (3000 mg/L) was diluted and assessed by immunoassay to be 3167 mg/L, 105.5% of the stated concentration. Dilute aqueous tobramycin solutions were applied to Schirmer test strips in 5–40 μl aliquots and extracted into drug-free human serum prior to tobramycin immunoassay to assess the linearity and recovery of the antibiotic (Figure 1). The extraction method recovered 96.5% of tobramycin from the Schirmer test strips (Figure 1). The within-day coefficient of variation to tear extraction from the Schirmer test strips was determined to be 6.5% at 0.86 mg/L and 1.7% at 6.8 mg/L (n = 8) while analyzing 20 μL samples (Figure 1). The limit of detection was estimated to be 0.06 mg/L in 20 μL samples.

### Tobramycin concentration in horses’ tears

**Figure 2:**
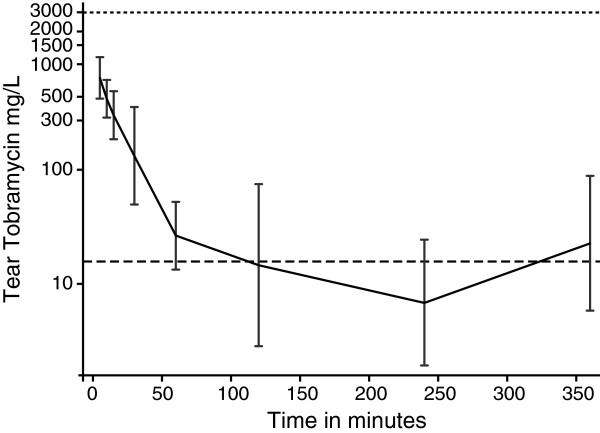
**Pharmacokinetics of tobramycin ophthalmic solution in horses’ tears:** The geometric mean concentration of tobramycin in tears was determined over 6 h (n = 5 at time points 5, 10, 15, 30 min and 1, 2, 4 and 6 h) after single topical administration of 0.3% tobramycin ophthalmic solution. Tobramycin concentrations were log transformed to derive the mean and standard deviation. Bars represent mean ± SD. Horizontal dashed lines are shown for the concentration of tobramycin in the ophthalmic solution, 3000 mg/L and the MIC_90_ of 16 mg/L.

The mean volume of tear fluid determined gravimetrically was 19 μL per Schirmer strip and tear volumes ranged from 4 to 37 μL. Tobramycin levels were determined in the drug-free human serum used to extract the Schirmer strips and the amount of tobramycin was expressed per volume of tear fluid to determine the tear drug concentration at each time point. The mean tobramycin concentrations at 5, 10, 15, and 30 minutes after administration were 759 (± 414), 489 (± 237), 346 (± 227), 147 (± 264) mg/L (Figure 2). The mean tobramycin concentration at 1, 2, 4, and 6 hours after administration were 27.6 (± 28.4), 14.8 (± 66.6), 6.7 (± 18.6), and 23.4 (± 73.4) mg/L (Figure 2).

### Pharmacokinetics

The initial concentration of the tobramycin at time 0 was calculated to be 905 mg/L and mean concentration declined to 16 mg/L (MIC_90_) by 68.5 min. The elimination rate constant in the first 60 minutes was 0.059 min^-1^ associated with a half-life of 11.7 minutes.

## Discussion

The results of this study are consistent with other tear film studies conducted in humans, horses and rabbits. We found that the concentration of drug in the tear film rapidly declined exponentially in the first 50 minutes, linear on logarithmic axes, and then plateaued and became variable as the limit of detection for the drug assay was approached. After one hour, the low tear drug levels were variable. Other tear film pharmacokinetic studies in horses, dogs, and rabbits, showed a large amount of variation of tear antimicrobial concentrations [8-10]. The authors suggested that this variation may be explained by inter-animal variation and low sample numbers [8,9]. Similar to these studies, we also had one sample in each the 5 min, 2 hr, 4 hr and 6 hr groups that were below the limit of quantification. The previous studies hypothesized that this occurred from not fully extracting the drug from the strips, from errors in the amount of drug in the syringe because of air, and from loss from blinking after the drop was given [8,9]. In the previous studies the animal’s other eye was normal, but in our study the 4 hr and 6 hr samples were from the same horse [8,9]. This is suggestive of sensitivity to the drug resulting in increased tearing.

We showed that the mean tobramycin in the tear film reached the MIC_90_ of 16 mg/L after 68.5 minutes, indicating that therapeutic concentrations of the drug were maintained for over an hour, compared to the 10 minutes reported in the rabbit tears [10]. Our result of an initial concentration of 905 mg/L of tobramycin at time 0 is in agreement with the estimates from a previous study on tear volume in horses [13]. In addition, when our results are compared to the same study, tear production is likely the main factor involved in tobramycin clearance from the surface of the eye [13]. The horses used in the study were normal, however it would be expected that tobramycin levels in the tear film of a horse with a corneal ulcer would decrease more quickly than a normal horse, as corneal ulceration causes increased tear production.

Tear production is also impacted by drugs, including sedatives and general anesthetics. A study in dogs found that sedation with xylazine alone did not impact tear production, butorphanol caused a mild decrease in tear production, while the combination of the two drugs significantly decreased tear production [14]. Another study showed that in horses intravenous xylazine did not affect tear production, while general anesthesia with halothane caused a decrease in tear production [15]. This suggests that the xylazine used for sedation in the study did not impact the tear production. It is possible that the combination of xylazine and butorphanol used in the fractious horse caused decreased tear production, and subsequent elevated concentrations of tobramycin in the tear film. When the individual measurements from this horse were examined, the concentrations were in the middle of the range compared to the other horses in the group. This suggests that it is unlikely that the sedation had a significant effect.

The MIC_90_ for gram positive and gram negative ocular isolates from humans has previously been reported as 16 mg/L for tobramycin [12], but there is limited published data regarding the aminoglycoside susceptibilities for bacteria isolated from horses [16,17]. For equine keratitis isolates tested by use of minimum inhibitory concentration methods for tobramycin, susceptibility criteria were <4 mg/L for susceptible, 8 mg/L for moderately susceptible, and >16 mg/L for resistant isolates [18].

Treatment of bacterial infections requires therapeutic levels of antimicrobials within the tissues. Maintaining therapeutic concentrations of drug could be achieved by improving drug retention in the tears, by using higher concentrations of the drug, and by more frequent drug administration [19,20]. In this sense, a study in rabbits found higher concentrations of tobramycin in the tears and within the ocular tissues when the drug was combined with xanthan gum in a product called TobraDex ST® (Alcon Laboratories Inc., Fort Worth, TX, USA) versus tobramycin alone [20]. Exposure to the tear pH causes the ionic bond between the xanthan gum and drug molecules to break, increasing the viscosity of the drop when it is within the tear film, thus prolonging the retention of the drug in the tears [20]. This study found that tear tobramycin concentration was 8 and 12.5 times higher in the formulation using xanthan gum 10 and 60 minutes after topical application respectively [20]. Interestingly, increasing retention in the tears did not affect the aqueous humour concentrations of the drugs [20].

Using higher drug concentrations is another way to achieve therapeutic levels within infected tissues. A study in rabbits demonstrated that tear and corneal tobramycin levels increased proportionally with the concentration of drug administered [19]. Tobramycin was not detected in the cornea or aqueous 15 minutes following application of a 0.3% solution and concentrations greater than 1.1% were required to achieve penetration into the aqueous. However, tobramycin concentrations higher than 0.3% caused a significant decrease in the rate of corneal healing following hourly drug application [19]. Further, the use of 1.1% and 4.0% tobramycin solutions administered hourly compared to 0.3% administered every 15 minutes did not statistically reduce the number of colony forming units (CFU) in *Pseudomonas aeruginosa* keratitis in rats [19]. Interestingly, an in vitro study showed that tobramycin affected the migration of canine corneal epithelial cells less than all the other antimicrobial agents tested, including ciprofloxacin [21]. This suggests that tobramycin has the least impact on corneal wound healing, making it potentially more advantageous in the treatment of superficial corneal ulcerations [21].

A recent study in horses examined the ocular penetration of ciprofloxacin and moxifloxacin through intact corneal epithelium following topical application [22]. Therapeutic levels of both drugs were found in the tear film and cornea, but similar to the tobramycin studies, aqueous humor concentrations of moxifloxacin and ciprofloxacin were low and undetectable, respectively [22]. Further research is needed to determine the penetration of tobramycin into the ocular tissues.

In addition to pharmacokinetics considerations, the development of antimicrobial resistance is a concern in both human and veterinary ophthalmology. While a study from Florida found that *Pseudomonas aeruginosa* showed a statistically significant increase in resistance to tobramycin over several years, other studies found that 100% of their cultures were susceptible to the drug [2,5]. In order to minimize the development of further antimicrobial resistance, tobramycin should be used judiciously or in combination with other antibiotics based on pharmacokinetics studies in each species [2,23].

## Conclusions

This study validated an extraction and immunoassay method allowing the measurement of tobramycin in equine tears. We are also describing for the first time the pharmacokinetics of tobramycin ophthalmic solution in horses. Pharmacokinetic information is vital to avoid empirical use of antibiotics and development of bacterial resistance. Further research is needed to determine the penetration of tobramycin into equine ocular tissues in order to aid in the development of therapeutic schedules.

## Abbreviations

MIC: Minimum inhibitory concentration; MIC_90_: Minimum inhibitory concentration required to inhibit the growth of 90% of organisms.

## Competing interest

The authors have no competing interests to declare.

## Authors’ contribution

SC participated in the design of the study, the clinical experiments, as well as the immunoassay analysis and drafted the manuscript. AL supervised the immunoassay analysis and drafted the manuscript. BS participated in the design of the study and the clinical experiments. RL conceived the study and participated in its design, the clinical experiments as well as the writing of the manuscript. All authors read and approved the final manuscript.
